# Autoimmune Diseases in Patients with Premature Ovarian Insufficiency—Our Current State of Knowledge

**DOI:** 10.3390/ijms22052594

**Published:** 2021-03-05

**Authors:** Anna Szeliga, Anna Calik-Ksepka, Marzena Maciejewska-Jeske, Monika Grymowicz, Katarzyna Smolarczyk, Anna Kostrzak, Roman Smolarczyk, Ewa Rudnicka, Blazej Meczekalski

**Affiliations:** 1 Department of Gynecological Endocrinology, Poznan University of Medical Sciences, 60-535 Poznan, Poland; annamaria.szeliga@gmail.com (A.S.); marzena@jeske.pl (M.M.-J.); ankos30@op.pl (A.K.); 2 Department of Gynaecological Endocrinology, Medical University of Warsaw, 00-315 Warsaw, Poland; calikowna@wp.pl (A.C.-K.); monika.grymowicz@wp.pl (M.G.); rsmolarczyk@poczta.onet.pl (R.S.); 3 Department of Dermatology and Venereology, Medical University of Warsaw, 00-315 Warsaw, Poland; ksmolarczyk@gmail.com

**Keywords:** POI, autoimmune, APS, Hashimoto disease

## Abstract

Premature ovarian insufficiency (POI), previously known as premature ovarian failure or premature menopause, is defined as loss of ovarian function before the age of 40 years. The risk of POI before the age of 40 is 1%. Clinical symptoms develop as a result of estrogen deficiency and may include amenorrhea, oligomenorrhea, vasomotor instability (hot flushes, night sweats), sleep disturbances, vulvovaginal atrophy, altered urinary frequency, dyspareunia, low libido, and lack of energy. Most causes of POI remain undefined, however, it is estimated that anywhere from 4–30% of cases are autoimmune in origin. As the ovaries are a common target for autoimmune attacks, an autoimmune etiology of POI should always be considered, especially in the presence of anti-oocyte antibodies (AOAs), autoimmune diseases, or lymphocytic oophoritis in biopsy. POI can occur in isolation, but is often associated with other autoimmune conditions. Concordant thyroid disorders such as hypothyroidism, Hashimoto thyroiditis, and Grave’s disease are most commonly seen. Adrenal autoimmune disorders are the second most common disorders associated with POI. Among women with diabetes mellitus, POI develops in roughly 2.5%. Additionally, autoimmune-related POI can also present as part of autoimmune polyglandular syndrome (APS), a condition in which autoimmune activity causes specific endocrine organ damage. In its most common presentation (type-3), APS is associated with Hashomoto’s type thyroid antibodies and has a prevalence of 10–40%. 21OH-Antibodies in Addison’s disease (AD) can develop in association to APS-2.

## 1. Premature Ovarian Insufficiency

Premature ovarian insufficiency (POI), previously known as premature ovarian failure or premature menopause, is defined by loss of ovarian function before the age of 40 years [1]. It is associated with hypoestrogenism and loss of residual follicles which leads to menstrual abnormalities, infertility, and decreased health-related quality of life [1,2]. The diagnosis of POI is based on the European Society of Human Reproduction and Embryology (ESHRE) criteria and includes: amenorrhea or oligomenorrhea for at least four months and an elevated follicle stimulating hormone (FSH) level > 25 IU/I on two occasions at least 4 weeks apart [1].

The average menopausal age in Caucasian women is approximately 51 years. Having menopause before 45 years of age is recognized as early menopause and occurs in about 5% of women. In perimenopause, the number of ovarian follicles decreases from 5–6 million (prenatally) to about 1000 in each ovary. Inferior egg quality is related to a sharper decline in the number of follicles [3].

The risk of POI before the age of 40 years is around 1% and its prevalence varies with age. Prevalence is 1:10,000 at the age of 18–25 years, 1:1000 at age 25–30 years, and 1:100 at 35–40 years [3,4]. The occurrence of POI depends also on ethnicity, with highest incidence in Caucasian, African American, and Hispanic women [5]. The frequency of POI among women with primary amenorrhea is 10–28%, whereas it is 4–18% in women with secondary amenorrhea. POI is a heterogenous disease which often remains undiagnosed. Most known causative factors are genetic, iatrogenic, infectious, or autoimmune [6].

Clinical symptoms of POI are largely the result of estrogen deficiency and may include amenorrhea, oligomennorhea, vasomotor instability (hot flushes, night sweats), sleep disturbances, vulvovaginal atrophy, altered urinary frequency and recurrent infections, mood disorders including irritability and emotional lability. Other hormonal imbalances, such as androgen insufficiency also have a marked influence on the symptomology in POI. Androgen deficiency manifests as low libido, dyspareunia, and lack of energy [4]. Women affected by POI are also at increased risk of cardiovascular disease, dementia, cognitive decline, Parkinsonism, and osteoporosis [2]. They very often require psychological counseling to address issues relating to the diagnosis, such as infertility, lower self-esteem, and increased rates of anxiety and depression [2,3].

To avoid long-term consequences of estrogen deficiency, hormone replacement therapy is recommended [1]. Women with POI should also be encouraged to maintain an active lifestyle, avoid or stop smoking, and sustain a healthy weight to help ameliorate the adverse effects of hypoestrogenism [1,2].

The clinical course of POI often includes intermittent and unpredictable ovarian function seen in up to 50% of patients even years after diagnosing. Intermittent spontaneous ovulation occurs in approximately 20% of women which a decreases in frequency over time. Between 5–10% of affected patients are able to conceive and carry pregnancy to delivery [6].

## 2. Causes of POI

The exists a wide variety of causes leading to POI. Potential etiologies can be subdivided into different groups: idiopathic, genetics, autoimmune; iatrogenic, and environmental. The cause of POI in the majority of cases remains unknown [7]. Idiopathic cases are reported is as high as 74–90% of POI patients.

The extent to which genetics play a role in the development of POI remains unclear. It is suggested that around 7% of cases are purely genetically-induced [8]. Much of our understanding is extrapolated from X chromosome abnormalities and role in ovarian dysgenesis. POI in women with Turner syndrome (45, X0), trisomy X (47, XXX), and tetrasomy X (48, XXXX) is widely observed [9]. Numerous genetic aberrations have correlated with POI, including genes on the X chromosome (such as bone morphogenetic protein 15 (BMP15), progesterone receptor membrane component I (PGRMC I), fragile mental retardation I (FMR I), androgen receptor (AR), and premature ovarian failure IB (POFIB). It is also linked to abnormalities and was also observed on autosomal chromosomes such a growth differentiation factor 9 (GDF9), folliculogenesis specific b HLH transcription factor (FIGLA), new ovary homeobox gene (NOBOX), estrogen receptor 1 (ESR1), FSH receptor (FSHR). More studies are necessary to find out the connection between occurrence of POI and genetic factors (Table 1).

Autoimmune-related diseases are estimated to drive anywhere from 4–30% of POI cases [10]. The most strongly associated group is that of thyroid-related disturbances such as hypothyroidism, Hashimoto thyroiditis, and Grave’s disease [11]. After thyroid diseases, the second most commonly associated group of autoimmune disorders is those relating to the adrenals. According to the literature, up to 10–20% of patients suffering with Addison’s disease will develop POI. Women with diabetes mellitus also at higher risk of developing POI with an estimated prevalence of 2.5%. POI has also been associated with numerous other disorders including rheumatoid arthritis, Crohn’s disease, myasthenia gravis, systemic lupus erythematosus, and multiple sclerosis [12].

Unfortunately, iatrogenic POI is often observed in patients who undergo treatment for malignancy. Patients with cancer in childhood and during adolescence are at higher risk of development POI in later life. POI in this group is most often a result of ovarian surgery, chemotherapy, and/or pelvic radiotherapy. Among those receiving chemotherapy, ovarian damage depends largely on the age of patient during treatment, chemotherapeutic agent(s) received, dose and duration of treatment [13]. Prepubertal patients show relative resistance to these effects of chemotherapy [13].

Finally, a not insignificant portion of POI is found to be caused by environmental factors. The most commonly associated environmental factor was found to be cigarette smoking and presents a significant risk factor for future POI [14]. Interestingly, there is a well-documented correlation in the literature between POI and incidence of recurrent viral infections [14].

## 3. Pathophysiological Basis of Autoimmune POI

POI was first clinically recognized in the 1930s. In 1942, Fuller Albright observed for the first time a case in which early menopausal changes were associated with adrenal insufficiency. To date, the etiology of most diagnosed cases of POI remains undefined. It is known that autoimmune diseases coexists in approximately 10–55% of patients with POI. It is assumed that autoimmune disorders trigger anywhere from 4 to 30% of cases of POI [10,11]. The ovary is a common target for autoimmune disease following activation by environmental or genetic factors. Associated autoimmune POI should always be considered in the presence of a primary diseases such as autoimmune thyroiditis, diabetes type 1, celiac disease, myasthenia gravis, systemic lupus erythematosus, or any family history of autoimmune diseases [4,15]. In these cases, POI is often associated with seropositive anti-oocyte antibodies (AOAs) or presence of lymphocytic oophoritis on biopsy. Reliable testing methods to aid in the diagnosis of autoimmune cases are still under development.

### 3.1. Lymphocytic Oophoritis

Autoimmune lymphocytic oophoritis presents with mononuclear infiltration of ovarian theca cells, specifically in developing follicles and corpus luteum while sparing primordial and primary follicles [11,16]. Ovaries in this stage can be normal in size or enlarged. On ultrasound, residual follicular structures can be seen in the ovaries. No reliable serum markers of autoimmune oophoritis are currently available on the market [17]. Diagnosis requires ovarian biopsy, which present inherent risks and of itself is not routinely recommended. Histopathological confirmation of disease is only undertaken in approximately 10% of POI cases [18]. Cellular, as well as in humoral immunity is often affected. Immunohistochemistry reveals a predominant T-lymphocyte (CD4+, CD8+) infiltration, with B-lymphocytes, natural killer cells, and plasma cells producing mostly IgG antibodies [11]. Changes in immune system response leads to macrophage and dendritic cell abnormalities. The CD4+/CD8+ ratio and expression of class II MHC antigens by granulosa cells are often abnormal. Conflicting research exists on this point with some reporting a responsive increase in CD4+/CD8+ ratio, while others deny any such change or even report a significant decrease in CD4+ and CD25+ Treg cells count [15,19]. The inflammatory response affects steroid producing cells in the preovulatory follicles, notably the internal and external layers of theca, corpora luteum, and occasionally also granulosa cells. Sometimes perineural and perivascular infiltration is seen [18,19]. Difficulties in establishing a diagnosis is thought to be due to the transient state and biphasic nature of POI which only leads to atrophy in its final stage [20]. This would explain the decrease in ovarian volume and recession of inflammatory signs seen in ovarian biopsies when POI is finally confirmed [21,22]. Some observational data suggest that immunosuppressive therapies demonstrating a favorable safety profile can be beneficial in restoring ovarian function. It is suggested that this approach may have a positive effect on delaying POI and on slowing its progression to late stages rather than to be used after follicular reserves are depleted. Nevertheless, no benefit was demonstrated with glucocorticosteroid or anti-B-cell therapies [4,18].

### 3.2. Anti-Oocyte Antibodies

Anti-oocyte antibodies (AOAs) are found in 24–73% of patients with confirmed POI [20,23]. Sources conflict in the accuracy of these findings and the exact prevalence remains unclear. It is worth noting that the presence of AOAs in other fertility-influencing conditions is associated with a poor overall response to IVF treatment [18]. AOAs are positive in almost one third of women with unexplained infertility. For this reason, it is recommended that every infertile woman be investigated for early stage autoimmune POI.

Numerous antibody types can be involved in ovarian insufficiency with a wide range of antigenic targets in the ovary. Most frequently, these are directed against gonadotropins and their receptors. The β-subunit of follicle stimulating hormone (FSH) is most often targeted [24]. Other antibodies can theoretically act against hormone-producing cells, corpus luteum, zona pellucida, granulosa cells, oocyte, MATER (maternal antigen that embryos require), steroidogenic enzymes such as aldehyde dehydrogenase-1A1 (ALDH1A1), selenium binding protein 1 (SBP1), human heat shock protein 90-β (HSP90β), 3β-hydroxysteroid dehydrogenase (3β-HSD), and anti-α-enolase [15,18]. Several other active antibodies have been found, such as anticardiolipin or antinuclear antibodies. The exact correlation between AOAs and FSH or inhibin B levels remains to be established. Such antibodies may often appear years before clinical symptoms of ovarian disturbance are first noticed. Their role, however, as a marker for POI is limited due to the low specificity of existing tests leading to a high rate of false positive results and lack of validation [23].

### 3.3. Other Autoimmune Diseases and Antibodies to Steroid-Producing Cells

Adrenal autoimmune disorders are the second most common disorders associated with POI. Among women with diabetes mellitus, POI develops in roughly 2.5%.

POI can occur in isolation, but is often associated with other autoimmune conditions. Concordant thyroid disorders such as hypothyroidism, Hashimoto thyroiditis, and Grave’s disease make up the most commonly seen associated disorders. Autoimmune-related POI can also present as part of autoimmune polyglandular syndrome (APS), a condition in which autoimmune activity causes specific endocrine organ damage. APS-2 is associated with Hashimoto’s thyroid antibodies and has a prevalence of 10–40%. 21OH-Antibodies in Addison’s disease (AD) can also develop in APS-2 [25].

The prevalence of POI developed during AD is 10–20% whereas the prevalence of antibodies to 21-hydroxylase/ adrenocortical antibodies (21OH-Abs/ACAs) in POI ranges from 2.5 to 20%. Usually POI precedes AD by 8–14 years [26]. Conversely, it is found that all women with biopsy-confirmed autoimmune oophoritis are also positive for ACAs [25,27,28].

APS-1 is caused by mutation in the AIRE gene, which regulates immunological tolerance. In APS-1, approximately 40–70% of women will develop POI [18,27]. This high prevalence of both AD and POI is a result of autoantibodies to steroid-producing cells (StCA). The antigenic factors targeted by StCAs include 17alpha hydroxylase/17,20-lyase (17-OH), P-450 side chain cleavage enzyme (P-450scc), 21-hydroxylase (21-OH), 3β-HSD antibodies [18,26]. StCAs can be found in APS-1, APS-2, AD, and POI in as many as 60%, 25–40%, 60–90%, and 3–10% of cases, respectively. In cases where POI and AD coexist, StCAs are found in 87–100% of women. This would suggest a promising marker for predicting ovarian insufficiency in autoimmune AD [28,29]. Although in isolated POI StCAs are rarely detected, various other organ-specific or non-organ specific antibodies such as ACA, ANA, and anti-ds-DNA are found in 34–92% of patients [29]. The role of StCAs in idiopathic POI remains undefined [30]. Other autoimmune diseases observed in association with POI include diabetes mellitus, hypophysitis, idiopathic thrombocytopenic purpura, vitiligo, alopecia, autoimmune hemolytic anemia, pernicious anemia, systemic lupus erythematosus (SLE), rheumatoid arthritis, Crohn’s disease, Sjogren’s syndrome, primary biliary cirrhosis, and chronic active hepatitis [3,11,28]. The prevalence rate of developing POI in patients with SLE is 0.6–43%. There are two possible mechanisms by which ovarian follicles are depleted: autoimmune oophoritis and use of gonadal cytotoxic drugs such as cyclophosphamide (CYC). The cytotoxic effect of these medications can be mitigated and complications prevented by using a gonadotropin releasing hormone agonist (GnRh-a) as a fertility preserving agent during treatment [31]. Studies have suggested that such preventative action can decrease the incidence rate of developing SLE treatment-associated POI to that of the general (CYC-naïve) population [32].

### 3.4. Viral Infections and Vaccination

There are single reports of POI developing following viral infections and some case reports connect quadrivalent anti-HPV vaccination with AOA formation [33]. Molecular mimicry, epitope spreading, polyclonal activation of B-lymphocytes, or bystander activation are supposed to be possible mechanisms in creating self-antigens. Association with the quadrivalent HPV-vaccine was statistically significant, however, no cases have been identified following vaccination with HPV9 or HPV2 vaccines [4,34]. Due to the small number of case reports, and their ambiguity in reporting data, these studies have been found to be of low quality and should be interpreted cautiously. Naleway et al. concluded that no significant risk of developing POI was found after immunization with HPV, TdaP, II, or menACWY vaccines [33].

## 4. POI-Associated Autoimmune Diseases

### 4.1. Thyroid Autoimmunity

Since the 1950s, investigators have noted that premature ovarian insufficiency is associated with autoimmune diseases. One of the first associations between thyroid autoimmunity and POI was drawn in 1972, when de Moraes Ruehsen first found anti-Tg antibodies in a POI patient [35]. Thyroid autoimmune disease, most commonly Hashimoto’s thyroiditis, is present in 14% to 32.7% of women with POI at initial diagnosis [22,36,37]. Data on the prevalence of individual autoimmune diseases associated with POI are inconsistent at best. Some publications identify autoimmune thyroiditis as the most common autoimmune disease associated with POI, while others claim Addison’s disease to be the most common.

In one of the first studies on association between POI and autoimmune thyroiditis, it was found that among 50 patients with POI, 18% had clinical evidence of an autoimmune thyroid disorder, and 10% had seroconverted thyroid autoantibodies [38]. In another study, Goswami R et al. found that 24.1% of POI patients had positive titers for TPO-antibody in comparison to 9% in healthy controls (this was statistically significant). Similarly, the frequency of clinical thyroid dysfunction was significantly higher in patients with POI (20.6%) than in controls [39]. Nevertheless, it must be emphasized, that according to the United States National Health and Nutrition Examination Survey, TPO-antibodies and Tg-antibodies are present in 14.6% and 13.8% of disease-free women, respectively [40].

Patients with POI suffer from decreased ovarian reserve. One well known and commonly used marker of ovarian reserve is AMH. Osuka S et al. found no significant difference in serum AMH levels between the TPOAb or TgAb-positive women and those who were antibody-double negative. Moreover, serum AMH levels were not found to correlate significantly with the concentration of TgAb or TPOAb. On the other hand, serum AMH correlated negatively with TSH levels in patients who were either positive for TPOAb or TgAb. This suggests, that thyroid disfunction may exacerbate decreased ovarian reserve [37].

ESHRE guidelines emphasize the necessity to screen for thyroid antibodies (TPO-Ab) in every women with POI. If the patient is found to be TPOAb positive, TSH should be followed annually. There is, however, no consensus on whether periodic serum TPOAb titer and/or TSH should be monitored in women with POI when these tests were initially negative [1].

Moreover, all patient who desire to conceive (spontaneously in early POI or otherwise after oocyte donation), should undergo thyroid function testing and receive appropriate treatment as needed. This is due to the detrimental effects thyroid dysfunction has on fetal neurocognitive development [1].

### 4.2. Adrenal Gland Autoimmunity

Premature ovarian insufficiency can also be associated with adrenal glands disorders. It develops from the presence of cross-reacting autoantibodies and their response against cells responsible for steroid production (steroid cell autoantibodies; SCA) in different systems [23]. SCAs are classified as polyclonal immunoglobin belonging to the IgG class.

According to Bakalov et al. [24], approximately 10–20% of women diagnosed with Addison‘s disease have POI [24]. On the other hand, 2.5–20.0% women with POI present some symptom of adrenal autoimmunity [41]. La Marca et al. [42] found, that around 5% of women with POI will have positive titers for SCA [42].

Generally, POI will present before the onset of clinical adrenal involvement. In some cases, POI may occur 8–14 years before the onset of Addison’s [22]. De Bellis et al. [28] studied 33 young women below the age of 40 who were diagnosed with subclinical-clinical autoimmune Addison’s disease from 1993 to 2004. Each patient in this study had normal menstrual cycles with ovulation at time of enrolment and were followed for 10 years. The long-term, time-related variations in SCA titers, ovarian function, and ovarian reserve were evaluated. The authors concluded that early elevated SCA titers reliably predicted subsequent development of autoimmune POI and therefore held important clinical value. Occasionally, the diagnostic time line is reversed. Adrenal insufficiency can develop in up to 50% of women with adrenal autoimmunity [43]. As this is a severe and often life-threatening condition, it is very important to maintain a low threshold for suspicion and that appropriate adrenal screening and work-up is performed in patients with recognized POI. It is particularly important to monitor adrenal function in those patients who are planning a pregnancy with the use of assisted reproduction methods. In women with Addison’s disease, SCA titers can be used as markers for prediction and risk stratification for developing POI [21].

According to Clinical Guidelines screening for 21OH-Ab (or alternatively adrenocortical antibodies (ACA)) should be considered in women with POI of unknown cause or if an immune disorder is suspected. POI patients with positive 21OH-Ab/ACA test should be referred to an endocrinologist for further testing of adrenal function and to rule out Addison’s disease [1]. The endocrinological workup should include assessment of ACTH, renin activity, and ACTH stimulation test.

Further consideration is needed regarding repeat and follow-up testing intervals of serum autoantibody titers. According to ESHRE Guidelines, a negative titer for serum 21OH-Ab/ACA in women with POI indicates that no further re-testing is needed later in life, unless signs or symptoms of endocrine disease develop [1].

### 4.3. APS and Associated POI

There are four types of autoimmune polyendocrine syndromes: APS types I-IV, which are characterized by autoimmunity against two or more endocrine organs. Autoimmune oophoritis is associated with APS type 1 and 2 and is responsible for 2–10% of POI cases.

Autoimmune polyglandular syndrome type one (APS-1), also known as autoimmune polyendocrinopathy-candidiasis-ectodermal dystrophy/dysplasia (APECED), is caused by a mutation in the autoimmune regulator gene (AIRE) located on chromosome 21 (Table 2). It encodes a nuclear transcription factor which is of importance in the thymus and may be involved in regulatory T cell selection and generation. It also regulates self-tolerance from T-cell attacks [11]. It is believed that 45–60% of women with AIRE mutations will develop POI [22]. APS-1 predominantly manifests as primary adrenal insufficiency, mucocutaneous candidiasis, and hypoparathyroidism. Nevertheless, it can also be associated with a variety of other diseases (Table 2).

APS type II, also known as Schmidt Carpenter syndrome, is an autosomal dominant disease, making it more prevalent compared to ASP-I. It manifests mainly as Addison’s disease and thyroid autoimmunity or type 1 diabetes. Among patients with APS-II, between 10 and 25 percent will go on to develop POI [20] (Table 2).

Autoimmune polyglandular syndrome type 3 (APS-3) is characterized by the occurrence of autoimmune thyroiditis accompanied by other organ (non-ovarian specific) autoimmune disorders in the absence of adrenal insufficiency (Table 2). Szlendak-Sauer et al. observed that autoimmune thyroid disease was presented in 33.7% of patients with POI who were diagnosed as APS-III [46]. APS-4 is diagnosed when Addison’s disease is associated with any other autoimmune diseases excluding those specified in APS-1, -2, or -3 [41].

The prevalence of POI associated with APS varies with each APS type. The highest incidence was observed in patients with APS-1 (>40%), followed by APS-4 (30%), and lowest in APS-2 (16%) [11].

In patients with coexisting Addison’s disease and POI, the development of Addison’s disease tends to precede that of POI. A similar pattern is observed with APS-1, and APS-4. Such findings underline the possible advantage of StCA antibody testing in patients diagnosed with APS-1 and APS-4. Such testing should help to stratify the risk for POI development in these patients [11]. StCA antibodies were detected in the majority of APS-1 patients who presented with POI (11 of 13; 84.6%) [11].

The coincident presence of ovarian and adrenal failure in autoimmune polyglandular syndromes emphasizes the need to assess for adrenal autoimmunity and evidence of adrenal insufficiency in all subjects with POI, even in the absence of pathognomonic APS type I or II features. The presence of adrenal antibodies (ACAs and/or CYP21Abs) are predictive of future clinical adrenal failure in 100% of children < 16 years who were tested and therefore should be measured in any adolescent with POI [44].

### 4.4. Rare Coexistence with Hypoparathyroidism and Type I Diabetes

Hypoparathyroidism has been associated with POI as well as other endocrine disorders such as hyphophysitis, autoimmune adrenal insufficiency, type 1 diabetes mellitus, autoimmune haemolytic anaemia, celiac disease, inflammatory bowel diseases, glomerulonephritis, Sjogren’s syndrome, and myasthenia gravis [47].

Bano G et al. described a case in which hypoparathyroidism was associated with POI. This pathogenesis of hypoparathyroidism is related to a defect in the G protein-coupled FSH and LH receptors. Due to the large number of receptors which are G-protein stimulated, hypoparathyroidism was accompanied by POI [48]. The study described a patent with PHPT and POI as a result of a balanced reciprocal translocation of 46X, t(X; 2) (q22; p13). PHPT was most likely a result of chromosome 2 involvement [49]. In this case, the patient presented with raised corrected serum calcium and elevated serum parathyroid hormone concentrations among other clinical symptoms of POI.

The prevalence of type 1 diabetes in POI patients, according to available reports in the literature, is estimated at 2.5% [50]. Type I diabetes is usually diagnosed before developing symptoms of POI, there is, however, currently no significant data which would indication the need for routine screening for concomitant diabetes in patients with POI [1].

Studies on the subject of diabetes as it relates to POI are reported in the literature. Kulaksizoglu et al. conducted a study in which 43 women with POI were tested for (among others) glucose tolerance and insulin levels. Women diagnosed with POI had higher glucose and insulin levels compared with the control group as well as higher levels of copper, lower levels of vitamin D, and HOMA-IR, which the authors identified as “traditional risk factors for diabetes” [51]. These results were confirmed in a secondary study, where authors compared the prevalence of diabetes mellitus in patients with POI against controls, reaching a similar conclusion [52].

## 5. Screening of POI Patients Regarding Other Autoimmune Diseases

Anti-Mullerian hormone (AMH) is considered a reliable tool in patients with other autoimmune diseases for recognition and risk assessment of developing POI. AMH is decreased in patients with autoimmune thyroiditis regardless of serum FSH, estradiol, or inhibin B levels [3,25,28].

The ESHRE recommends routine screening for the presence of thyroid autoantibodies and 21OH-Abs/ACAs in every case of POI [1]. Some studies suggest confirming adrenal insufficiency using ACTH stimulation tests rather than only morning serum cortisol level [18]. Screening for anti-oocyte antibodies is not recommended because of the multiplicity of potential autoantigens in ovarian tissue. Moreover, the prevalence of AOA among POI patients varies greatly in the literature. Screening beyond Hashimoto’s and Addison’s diseases is not routinely performed [1]. The patient and clinician should, however, be aware of the higher risk for autoimmune processes in POI, and remain vigilant for concerning signs and clinical development of autoimmune diseases so as to allow for early diagnosis and treatment.

## 6. Conclusions

The risk of developing POI before the age of 40 years is 1%. Most causes of POI remain undefined, however, it is assumed that autoimmune processes account for 4 to 30% of cases. The ovary is a common target for autoimmune attack and an autoimmune etiology of POI should be considered in the presence of anti-oocyte antibodies (AOAs), autoimmune diseases, or lymphocytic oophoritis in biopsy.

POI can be isolated, but is often associated with other autoimmune conditions. It is essential to take into consideration other autoimmune pathologies whenever POI is recognized or suspected, and appropriate screening should be implemented.

## Figures and Tables

**Table 1:** Genetic causes of premature ovarian insufficiency (POI).

Genes	Chromosomal Localization
Turner syndrome	45 X0, Mosaicism (46XX/45X0; 46XY/45X0), Isochromosome Xq, Ring chromosome, Xp or Xq deletion
FMR1	Xq27.3
FMR2	Xq28
PGRMC1	Xq22–24
BMP	15Xp11.2
GDF9	5q31.1
FIGLA	2q13.3
LHR	2p21
FSHR	2p16–p21
FOXL	23q23
FOX03	6q21
SOHLH1	9q34.2
SOHLH2	13q13.3
NR5A1	9q33
WT1	11p13
MCM8	20p12.3
CSB-PGBD3	10q11.23
INHA	2q33–q36
ERα	6q25
SF1	11q13
ERβ	14q23.2
CYP19A1	15q21.1

**Table 2:** Autoimmune polyendocrine syndromes (APS) associated with POI [22,44,45].

APS-I/APECED	APS-II	APS-III
Autoimmune polyendocrinopathy-candidiasis-ectodermal dystrophy	Schmidt Carpenter syndrome	
Mucocutaneous candidiasis (75%) Addison’s disease (60–79%) Hypoparathyroidism (89%) Chronic active hepatitis Pernicious anemia Vitilgo Hypogonadism Type 1 diabetes mellitus Malabsorption syndrome Grave’s disease Autoimmune hypothyroidism IgA deficiency Alopecia Asplenism Ectodermal dysplasia Keratitis Pure Red Cell Aplasia	Addison’s disease Pernicious anemia Vitilgo Hypogonadism Type 1 diabetes mellitus Celiac disease dermatitis herpetiformis Grave’s disease Autoimmune thyroid disease IgA deficiency Alopecia Myasthenia gravis Idiopathic thrombocytopenia Parkinson’s disease Serositis Stiff-man syndrome Idiopathic Heart Block Hypophysitis	Autoimmune thyroid disease, and in addition: in APS-3A—type 1 diabetes mellitus, in APS-3B—pernicious anemia, in APS-3C—albinism and/or alopecia, or autoimmune diseases of other organs (e.g., celiac disease, hypogonadism, myasthenia gravis).
39–72% develop POI	10–25% develop POI	

## References

[1] Webber L., Davies M., Anderson R., Bartlett J., Braat D., Cartwright B., Cifkova R., Keizer-Schrama S.D.M., Hogervorst E., Janse F. (2016). ESHRE Guideline: Management of women with premature ovarian insufficiency. Hum. Reprod..

[2] Faubion S.S., Kuhle C.L., Shuster L.T., Rocca W.A. (2015). Long-term health consequences of premature or early menopause and considerations for management. Climacteric.

[3] Luisi S., Orlandini C., Regini C., Pizzo A., Vellucci F., Petraglia F. (2015). Premature ovarian insufficiency: From pathogenesis to clinical management. J. Endocrinol. Investig..

[4] Domniz N., Meirow D. (2019). Premature ovarian insufficiency and autoimmune diseases. Best Pr. Res. Clin. Obstet. Gynaecol..

[5] Luborsky J., Meyer P., Sowers M., Gold E., Santoro N. (2003). Premature menopause in a multi-ethnic population study of the menopause transition. Hum. Reprod..

[6] Jiao X., Zhang H., Ke H., Zhang J., Cheng L., Liu Y., Qin Y., Chen Z.-J. (2017). Premature Ovarian Insufficiency: Phenotypic Characterization within Different Etiologies. J. Clin. Endocrinol. Metab..

[7] Laven J.S.E. (2016). Primary Ovarian Insufficiency. Semin. Reprod. Med..

[8] Rossetti R., Ferrari I., Bonomi M., Persani L. (2017). Genetics of primary ovarian insufficiency. Clin. Genet..

[9] Reindollar R.H. (2011). Turner Syndrome: Contemporary Thoughts and Reproductive Issues. Semin. Reprod. Med..

[10] Kirshenbaum M., Orvieto R. (2019). Premature ovarian insufficiency (POI) and autoimmunity-an update appraisal. J. Assist. Reprod. Genet..

[11] Sharif K., Watad A., Bridgewood C., Kanduc D., Amital H., Shoenfeld Y. (2019). Insights into the autoimmune aspect of premature ovarian insufficiency. Best Pr. Res. Clin. Endocrinol. Metab..

[12] Collins G., Patel B., Thakore S., Liu J. (2017). Primary Ovarian Insufficiency: Current Concepts. South. Med. J..

[13] Gargus E., Deans R., Anazodo A., Woodruff T.K. (2018). Management of Primary Ovarian Insufficiency Symptoms in Survivors of Childhood and Adolescent Cancer. J. Natl. Compr. Cancer Netw..

[14] Vabre P., Gatimel N., Moreau J., Gayrard V., Picard-Hagen N., Parinaud J., Léandri R. (2017). Environmental pollutants, a possible etiology for premature ovarian insufficiency: A narrative review of animal and human data. Environ. Health.

[15] Kobayashi M., Nakashima A., Yoshino O., Yoshie M., Ushijima A., Ito M., Ono Y., Shima T., Kawamura K., Ishizuka B. (2019). Decreased effector regulatory T cells and increased activated CD4+ T cells in premature ovarian insufficiency. Am. J. Reprod. Immunol..

[16] Sedmak D.D., Hart W.R., Tubbs R.R. (1987). Autoimmune oophoritis: A histopathologic study of involved ovaries with immunologic characterization of the mononuclear cell infiltrate—PubMed. Int. J. Gynecol. Pathol..

[17] Irvine W.J. (1964). Thyroid Auto-Immunity as a Disorder of Immunological Tolerance. Q. J. Exp. Physiol. Cogn. Med. Sci..

[18] Ebrahimi M., Akbari A.F. (2015). The role of autoimmunity in premature ovarian failure—PubMed. Iran. J. Reprod. Med..

[19] Xiong J., Tan R., Wang W., Wang H., Pu D., Wu J. (2020). Evaluation of CD4+CD25+FOXP3+ regulatory T cells and FOXP3 mRNA in premature ovarian insufficiency. Climacteric.

[20] Hoek A., Schoemaker J., Drexhage H.A. (1997). Premature Ovarian Failure and Ovarian Autoimmunity*. Endocr. Rev..

[21] Silva C.A., Yamakami L.Y.S., Aikawa N.E., Araujo D.B., Carvalho J.F., Bonfá E. (2014). Autoimmune primary ovarian insufficiency. Autoimmun. Rev..

[22] Komorowska B. (2016). Autoimmune premature ovarian failure. Menopausal Rev..

[23] Pra C.D., Chen S., Furmaniak J., Smith B.R., Pedini B., Moscon A., Zanchetta R., Betterle C. (2003). Autoantibodies to steroidogenic enzymes in patients with premature ovarian failure with and without Addison’s disease. Eur. J. Endocrinol..

[24] Bakalov V., Vanderhoof V., Bondy C., Nelson L. (2002). Adrenal antibodies detect asymptomatic auto-immune adrenal insufficiency in young women with spontaneous premature ovarian failure. Hum. Reprod..

[25] Saglam F., Onal E.D., Ersoy R., Koca C., Ergin M., Erel O., Cakir B. (2014). Anti-Müllerian hormone as a marker of premature ovarian aging in autoimmune thyroid disease. Gynecol. Endocrinol..

[26] Magen E., Masalha A., Zueva E., Vardy D.A. (2018). Possible Autoimmune Primary Ovarian Insufficiency in Patients with Selective IgA Deficiency. Isr. Med. Assoc. J. IMAJ.

[27] Tang V.W., Faiman C. (1983). Premature ovarian failure: A search for circulating factors against gonadotropin receptors. Am. J. Obstet. Gynecol..

[28] De Bellis A., Bellastella G., Falorni A., Aitella E., Barrasso M., Maiorino M.I., Bizzarro E., Bellastella A., Giugliano D., Esposito K. (2017). Natural history of autoimmune primary ovarian insufficiency in patients with Addison’s disease: From normal ovarian function to overt ovarian dysfunction. Eur. J. Endocrinol..

[29] Luborsky J. (2002). Ovarian Autoimmune Disease and Ovarian Autoantibodies. J. Women’s Heal. Gender-Based Med..

[30] Novosad J.A., Kalantaridou S.N., Tong Z.-B., Nelson L.M. (2003). Ovarian antibodies as detected by indirect immunofluorescence are unreliable in the diagnosis of autoimmune premature ovarian failure: A controlled evaluation. BMC Women’s Health.

[31] Akawatcharangura P., Taechakraichana N., Osiri M. (2015). Prevalence of premature ovarian failure in systemic lupus erythematosus patients treated with immunosuppressive agents in Thailand. Lupus.

[32] Mayorga J., Alpízar-Rodríguez D., Prieto-Padilla J., Romero-Díaz J., Cravioto M.C. (2016). Prevalence of premature ovarian failure in patients with systemic lupus erythematosus. Lupus.

[33] Gong L., Ji H.-H., Tang X.-W., Pan L.-Y., Chen X., Jia Y.-T. (2020). Human papillomavirus vaccine-associated premature ovarian insufficiency and related adverse events: Data mining of Vaccine Adverse Event Reporting System. Sci. Rep..

[34] Naleway A.L., Mittendorf K.F., Irving S.A., Henninger M.L., Crane B., Smith N., Daley M.F., Gee J. (2018). Primary Ovarian Insufficiency and Adolescent Vaccination. Pediatrics.

[35] Ruehsen M.D.M., Blizzard R.M., Garcia-Bunuel R., Jones G.S. (1972). Autoimmunity and ovarian failure. Am. J. Obstet. Gynecol..

[36] Grossmann B., Saur S., Rall K., Pecher A.-C., Hübner S., Henes J., Henes M. (2019). Prevalence of autoimmune disease in women with premature ovarian failure. Eur. J. Contracept. Reprod. Health Care.

[37] Osuka S., Iwase A., Goto M., Takikawa S., Nakamura T., Murase T., Kato N., Kotani T., Kikkawa F. (2018). Bayasula Thyroid Autoantibodies do not Impair the Ovarian Reserve in Euthyroid Infertile Women: A Cross-Sectional Study. Horm. Metab. Res..

[38] Betterle C., Rossi A., Pria S.D., Artifoni A., Pedini B., Gavasso S., Caretto A. (1993). Premature ovarian failure: Autoimmunity and natural history. Clin. Endocrinol..

[39] Goswami R., Marwaha R.K., Goswami D., Gupta N., Ray D., Tomar N., Singh S. (2006). Prevalence of Thyroid Autoimmunity in Sporadic Idiopathic Hypoparathyroidism in Comparison to Type 1 Diabetes and Premature Ovarian Failure. J. Clin. Endocrinol. Metab..

[40] Hollowell J.G., Staehling N.W., Flanders W.D., Hannon W.H., Gunter E.W., Spencer C.A., Braverman L.E. (2002). Serum TSH, T4, and Thyroid Antibodies in the United States Population (1988 to 1994): National Health and Nutrition Examination Survey (NHANES III). J. Clin. Endocrinol. Metab..

[41] Reato G., Morlin L., Chen S., Furmaniak J., Smith B.R., Masiero S., Albergoni M.P., Cervato S., Zanchetta R., Betterle C. (2011). Premature Ovarian Failure in Patients with Autoimmune Addison’s Disease: Clinical, Genetic, and Immunological Evaluation. J. Clin. Endocrinol. Metab..

[42] La Marca A., Brozzetti A., Sighinolfi G., Marzotti S., Volpe A., Falorni A. (2010). Primary ovarian insufficiency: Autoimmune causes. Curr. Opin. Obstet. Gynecol..

[43] Forges T., Monnier-Barbarino P., Faure G.C., Bene M.C. (2004). Autoimmunity and antigenic targets in ovarian pathology. Hum. Reprod. Updat..

[44] Welt C.K. (2008). Autoimmune Oophoritis in the Adolescent. Ann. N. Y. Acad. Sci..

[45] Lewiński A., Ewa Płaczkiewicz-Jankowska E. (2019). APS-III. Internal Medicine.

[46] Szlendak-Sauer K., Jakubik D., Kunicki M., Skórska J., Smolarczyk R. (2016). Autoimmune polyglandular syndrome type 3 (APS-3) among patients with premature ovarian insufficiency (POI). Eur. J. Obstet. Gynecol. Reprod. Biol..

[47] Kumar N., Manesh I. (2017). Premature Ovarian Insufficiency: Aetiology and Long-Term Consequences. Women’s Health Open J..

[48] Ebrahimi M., Asbagh F.A. (2011). Pathogenesis and Causes of Premature Ovarian Failure: An Update. Int. J. Fertil. Steril..

[49] Bano G., Mansour S., Nussey S. (2008). The association of primary hyperparathyroidism and primary ovarian failure: A de novo t(X; 2) (q22p13) reciprocal translocation. Eur. J. Endocrinol..

[50] Podfigurna-Stopa A., Czyzyk A., Grymowicz M., Smolarczyk R., Katulski K., Czajkowski K., Meczekalski B. (2016). Premature ovarian insufficiency: The context of long-term effects. J. Endocrinol. Investig..

[51] Kulaksizoglu M., Ipekci S.H., Kebapcilar L., Kebapcilar A.G., Korkmaz H., Akyurek F., Baldane S., Gonen M.S. (2013). Risk Factors for Diabetes Mellitus in Women with Primary Ovarian Insufficiency. Biol. Trace Element Res..

[52] Gunning M.N., Meun C., Van Rijn B.B., Maas A.H.E.M., Benschop L., Franx A., Boersma E., Budde R.P.J., Appelman Y., Lambalk C.B. (2019). Coronary artery calcification in middle-aged women with premature ovarian insufficiency. Clin. Endocrinol..

